# Stress-Induced Transient Diabetic Ketoacidosis (DKA) in Pregnancy Without Pre-existing Diabetes

**DOI:** 10.7759/cureus.99575

**Published:** 2025-12-18

**Authors:** Megha Gupta, Shipra Tawade, Ahmed Shah Nazari, Ritesh Joshi

**Affiliations:** 1 Obstetrics and Gynaecology, Northampton General Hospital, Northampton, GBR

**Keywords:** diabetic ketoacidosis, gestational diabetes, intrauterine foetal demise, pregnancy, type i diabetes

## Abstract

Diabetic ketoacidosis (DKA) is a rare condition that can result in adverse maternal and foetal outcomes. Recognising atypical presentations such as euglycaemic or starvation ketoacidosis is crucial, as delayed diagnosis can be fatal. We report the case of a pregnant woman under 20 weeks’ gestation who presented with vomiting, dehydration, and metabolic acidosis. Laboratory findings revealed significant ketonaemia and acidaemia, with only transient hyperglycaemia lasting less than 24 hours. Her HbA1c was normal, diabetes antibodies were negative, and glucose monitoring remained normal throughout hospitalisation and subsequent follow-up, confirming the absence of underlying latent diabetes. The episode was likely precipitated by infection, dehydration, and starvation. Pregnancy amplifies the metabolic response to stress and starvation, lowering the threshold for ketone production. This case highlights the diagnostic challenge of recognising DKA physiology in non-diabetic pregnant patients.

## Introduction

Diabetic ketoacidosis (DKA) is a serious obstetric emergency characterised by the triad of hyperglycaemia, ketosis, and metabolic acidosis and is associated with significant maternal and foetal morbidity. It occurs most commonly in women with type 1 diabetes, less frequently in those with gestational or type 2 diabetes, and is exceptionally rare in women with normal glucose tolerance [1-4]. Pregnancy itself predisposes individuals to ketoacidosis through physiological insulin resistance, enhanced lipolysis, and altered carbohydrate metabolism. As a result, DKA may develop at lower or even near-normal glucose levels in pregnancy, making the diagnosis more challenging [5]. Precipitating factors include intercurrent infections, dehydration, medical or surgical stress, and medication non-compliance [1].

A related entity, starvation ketoacidosis, can occur in non-diabetic pregnant women following periods of inadequate caloric intake. This occurs because pregnancy increases ketone body production by approximately two- to four-fold compared with the non-pregnant state [6,7]. Another uncommon presentation is euglycaemic DKA (EDKA), defined as significant metabolic acidosis with near-normal blood glucose levels, which can further obscure timely recognition [8].

Given the potential for atypical biochemical profiles and subtle clinical manifestations, delayed diagnosis remains a considerable concern. Prompt identification and early initiation of therapy are critical to optimising maternal and foetal outcomes. Here, we present a unique case of DKA in pregnancy occurring in the context of transient hyperglycaemia lasting less than 24 hours and normal HbA1c levels, likely triggered by dehydration or sepsis. This case highlights the diagnostic difficulties posed by atypical presentations of DKA in pregnancy.

## Case presentation

A 30-year-old Caucasian woman, 17 weeks pregnant (gravida 4, para 2), attended the Accident and Emergency Department with a one-week history of epigastric pain, nausea, multiple episodes of vomiting, watery vaginal loss, and per-vaginal bleeding. The vomiting was so severe that the patient could not keep any solids or liquids down. She was unbooked and had not received any antenatal care. A private ultrasound scan performed a week ago had shown an intrauterine live pregnancy corresponding to 16 + 6 weeks of gestation.

Her past medical history included infective endocarditis and mitral valve repair six months earlier. She was also known to have hypertension, depression, and a history of alcohol abuse. However, in this episode, she gave no history of alcohol intake. There was no history of any drug abuse. The patient did not have any personal history of diabetes or gestational diabetes in her previous pregnancies, and no family history of diabetes. Her regular medications included aspirin, warfarin, labetalol, and sertraline.

On examination, she appeared prostrate and dehydrated, with dry mucous membranes suggestive of significant volume depletion. Her vital signs were a heart rate of 120 bpm, respiratory rate 20/min, oxygen saturation 100% on room air, temperature 36.5 °C, and blood pressure 118/78 mmHg. On auscultation, the chest was clear, and the cardiovascular examination was unremarkable. Abdominal examination revealed epigastric and suprapubic tenderness. Her Glasgow Coma Scale score was 15/15. On gynaecological examination, preterm pre-labour rupture of membranes (PPROM) was confirmed.

A venous blood gas (VBG) analysis showed a pH of 7.034, glucose 15.6 mmol/L, lactate 3.1 mmol/L, base excess −25.9, ketones 5.6 mmol/L (reference 0.6-1.5 mmol/L), and bicarbonate 8.2 mmol/L, consistent with severe metabolic acidosis, hyperglycaemia, and ketosis.

The laboratory investigations are charted in Table 1.

**Table 1:** Serial laboratory parameters, follow-up results, and reference ranges

Parameter	Units	Day 1	Day 2	Day 3	One-month / three-month follow-up	Reference range (adult female)
Haemoglobin	g/L	153	101	102	—	115–155
Platelet count	×10⁹/L	586	287	259	—	150–400
White blood cells	×10⁹/L	33.5	15.5	6.4	—	4.0–10.0
eGFR	mL/min/1.73 m²	71	>90	>90	—	>90
Serum glucose	mmol/L	15.6	—	—	—	3.9–5.8 (fasting)
Creatinine	µmol/L	93	64	44	—	45–84
Sodium	mmol/L	137	136	141	—	133–146
Potassium	mmol/L	5.0	4.4	3.2	—	3.5–5.3
Chloride	mmol/L	109	—	—	—	95–108
AKI stage	—	2	0	0	—	0
Insulin antibody	—	Negative	—	—	—	Negative
Anti-GAD antibody	—	Negative	—	—	—	Negative
HbA1c	mmol/mol	31	—	—	26 / 31	20–41

A bedside trans-abdominal ultrasound revealed anhydramnios and absent fetal cardiac activity, confirming intrauterine demise.

The diagnosis of DKA was established, as the diagnostic criteria were met (blood ketones > 3 mmol/L, pH ≤ 7.3, blood glucose > 11 mmol/L). This is clearly indicated in Tables 1-2, according to which this patient's blood sugar was 15.6 mmol/l on admission, pH was 7.034, and ketone levels were 5.6 mol/l. The diabetes team reviewed the patient and advised initiation of the DKA management protocol, including two-hourly monitoring of blood glucose and ketone levels and ordering of HbA1c and diabetes antibody tests.

Management was commenced with 0.9% sodium chloride maintenance fluids, variable-rate insulin infusion, and antiemetic therapy. The aim of treatment was to restore circulation, correct electrolyte disturbances, normalise ketone levels, and manage the precipitating cause. Serial VBGs were used to monitor the trends in acidosis and lactate. Blood ketones normalised within a few hours of treatment, and blood glucose levels also returned to normal (Table 2).

**Table 2:** Serial venous blood gas and metabolic parameters with reference ranges

Parameter	Units	On admission	Four hours later	Eight hours later	10 hours later	14 hours later	20 hours later	Reference range
pH	—	7.034	7.101	7.123	7.213	7.248	7.351	7.35–7.45
Glucose	mmol/L	16	8.8	9.2	6.4	7.5	6.2	3.9–5.8 (fasting)
Lactate	mmol/L	3.1	1.2	1.8	1.7	1.8	1.5	0.5–2.2
Base excess	mmol/L	-25.9	-22.9	—	-15.7	-12.3	-11.8	-2 to +2
Ketones	mmol/L	5.6	5.6	3.5	Normal	<0.1	N	0.0–0.6
Bicarbonate	mmol/L	8.2	9.4	—	13.2	14.6	16.1	22–29

The patient was also started on the sepsis pathway. Empirical intravenous meropenem was initiated, and blood cultures were sent, which subsequently returned negative. The DKA protocol was discontinued after approximately 12 hours, and she was switched to Levemir 10 units once daily with as-needed NovoRapid insulin.

On the following day, she spontaneously passed the foetus and was given medical management with misoprostol for retained products of conception. The patient’s clinical condition improved, and her blood glucose levels remained within the normal range, not requiring any further insulin during the next three days. Her investigations also improved significantly (Table 1).

The diabetic team re-evaluated her and considered possible gestational diabetes, although she required no insulin support. Her HbA1c was 31 mmol/mol (within normal range), and diabetes antibodies were negative. The patient was discharged on day 5 with a course of antibiotics and was advised to continue home blood glucose monitoring. She was scheduled for a follow-up appointment in the outpatient diabetes clinic.

At the three-month follow-up, she was clinically well, and all home blood glucose readings remained within the normal range. The endocrinologist concluded that this represented an unusual case of stress-induced transient hyperglycaemia rather than true diabetes mellitus. A glucose tolerance test (GTT) performed during her subsequent pregnancy was normal.

## Discussion

DKA is a rare condition; however, delays in diagnosis and management can be life-threatening for both mother and foetus. According to one case study, the perinatal mortality associated with DKA was reported to be 16% [1]. This is consistent with our case, in which the patient experienced a miscarriage at presentation.

Pregnancy is associated with complex metabolic changes, including increased insulin resistance mediated by hormones such as human placental lactogen, cortisol, and progesterone. These changes predispose pregnant women to an accelerated response to starvation, resulting in increased fat breakdown and fatty-acid flux to the liver, and rapidly progressing ketosis even in the absence of diabetes [9]. In one case report, a 27-week pregnant patient developed severe euglycemic ketoacidosis following appendicectomy, precipitated by prolonged perioperative fasting and vomiting at presentation, despite no prior history of diabetes. This is comparable to our case, where infection, dehydration, and starvation resulted in metabolic stress and produced a DKA-like biochemical picture despite the absence of underlying diabetes. However, our case is unique because of associated hyperglycaemia, which is not a typical feature of starvation ketoacidosis [9].

Another case report described ketoacidosis due to starvation caused by repeated vomiting in a pregnant woman with severe COVID-19 pneumonia [10]. Stress from the COVID-19 infection, along with elevated pro-inflammatory markers, contributed to increased lipolysis and ketone production. Similarly, in our case report, the patient had raised white blood cell counts and was treated as suspected sepsis with intravenous antibiotics, supporting the diagnosis of stress-related ketoacidosis.

In this case report, the patient was thoroughly evaluated for diabetes; however, her HbA1c remained normal throughout follow-up, including at three months postpartum, effectively ruling out pre-existing diabetes. Diabetes autoantibodies were negative, and home glucose monitoring remained within normal range. Furthermore, her glucose tolerance test in a subsequent pregnancy was normal. All these findings support that the patient was never diabetic and that her ketoacidosis was precipitated by infection and dehydration. Similarly, in one case series, three pregnant patients developed DKA but had only minimal elevations in HbA1c, negative islet-cell antibodies, and normal glucose tolerance tests performed at 24-28 weeks of pregnancy. As in our case report, all three patients in this case series had elevated infection markers [11]. Another case report also described the development of DKA in a 29-week pregnant patient with normal HbA1c who experienced foetal demise and was managed with fluid replacement, insulin, and antibiotics for a urinary tract infection [12].

This case highlights the importance of maintaining a high index of suspicion for DKA in pregnant patients presenting with dehydration or underlying infection, even in the absence of a history of diabetes. It is particularly notable because the hyperglycaemia was transient, with the patient requiring insulin therapy for only 24 hours. It is essential to always check ketones and venous blood gas in pregnant women who present with vomiting, dehydration, or tachypnoea, regardless of diabetic status. Early recognition and prompt management with fluid resuscitation, insulin therapy, and infection control are critical to preventing life-threatening acidosis.

## Conclusions

This case report highlights the development of ketoacidosis in a non diabetic pregnant patient precipitated by the stress of sepsis and dehydration, managed well as per protocol for the management of DKA.

DKA is a life-threatening emergency that carries significant risk for both mother and foetus. Prompt diagnosis and treatment are essential to reducing morbidity in the mother and preventing adverse foetal outcomes. However, recognising the possibility of DKA in a non-diabetic pregnant woman can be particularly challenging, especially in pregnancies under 20 weeks’ gestation. Regardless of the underlying aetiology, the management principles remain the same and include intravenous fluids, insulin infusion, and correction of acidosis and ketonemia.

This case underscores the importance of maintaining a high index of suspicion for DKA even in non-diabetic pregnant patients who present unwell, particularly when precipitating factors such as sepsis, dehydration, or foetal demise are present. Stress, infection, or starvation can transiently unmask DKA-like metabolic physiology in women without underlying diabetes. Post-recovery follow-up is essential to ensure complete resolution and to exclude latent or evolving diabetes.
